# A Data Acquisition Protocol for a Reactive Wireless Sensor Network Monitoring Application

**DOI:** 10.3390/s150510221

**Published:** 2015-04-30

**Authors:** Femi A. Aderohunmu, Davide Brunelli, Jeremiah D. Deng, Martin K. Purvis

**Affiliations:** 1 Information Science Department, University of Otago, Dunedin 9016, New Zealand; E-Mails: jeremiah.deng@otago.ac.nz (J.D.D.); martin.purvis@otago.ac.nz (M.K.P.); 2 National Inter-University Consortium for Telecommunications (CNIT), Pisa 56124, Italy; 3 Department of Industrial Engineering (DII), University of Trento, Povo I-38123, Italy; E-Mail: davide.brunelli@unitn.it

**Keywords:** wireless sensor network, communication reduction, compressive sensing, data acquisition protocol, environmental monitoring networks

## Abstract

Limiting energy consumption is one of the primary aims for most real-world deployments of wireless sensor networks. Unfortunately, attempts to optimize energy efficiency are often in conflict with the demand for network reactiveness to transmit urgent messages. In this article, we propose SWIFTNET: a reactive data acquisition scheme. It is built on the synergies arising from a combination of the data reduction methods and energy-efficient data compression schemes. Particularly, it combines compressed sensing, data prediction and adaptive sampling strategies. We show how this approach dramatically reduces the amount of unnecessary data transmission in the deployment for environmental monitoring and surveillance networks. SWIFTNET targets any monitoring applications that require high reactiveness with aggressive data collection and transmission. To test the performance of this method, we present a real-world testbed for a wildfire monitoring as a use-case. The results from our in-house deployment testbed of 15 nodes have proven to be favorable. On average, over 50% communication reduction when compared with a default adaptive prediction method is achieved without any loss in accuracy. In addition, SWIFTNET is able to guarantee reactiveness by adjusting the sampling interval from 5 min up to 15 s in our application domain.

## 1. Introduction

Wireless sensor networks (WSNs) are designed to provide information about the environment they are deployed to sense. They are typically characterized by a limited energy budget, because they are battery-powered. In recent years, wireless sensor networks have continuously gained attention, particularly due to their increasing capabilities to sense, store, process, and communicate information at low cost. For example, commercial sensor nodes such as the TelosB from Memsic [1], as well as W24TH from WISPES [2], and the software platforms from TinyOs [3], have facilitated the building of real-world testbeds both for convenient testing and for the evaluation of various application scenarios. The demand for the use of WSNs for applications such as health, habitat, seismic and wildfire monitoring has driven the growth of WSNs over the years partly because of the new era of Internet-of-Things (IoT), which is currently pushing the growth of the machine-to-machine (M2M) model of communication between several heterogeneous devices. We can expect that the use of WSNs in these fields will grow dramatically in the coming years.

Most implementations of WSNs are driven by different application-specific needs. The majority of the current real-world deployments have been applied to event detection systems. Events as used in this context may range from comparatively simple detections, such as changes in micro-climate condition in a home environment, wildfire monitoring, *etc.*, to more sophisticated scenarios such as intrusion detection, patient physical movement in a hospital *etc*. The authors in [4] (p. 441) reviewed two main approaches to tackle event detection problems, in particular they discussed extensively an algorithmic approach, which includes threshold usage, pattern recognition, and anomaly detection methods. One of the key characteristics of these methods is that they all leverage on the data processing capability of the sensor nodes to locally extract semantically meaningful information from the sensed data. Overall their goal was to limit the energy consumption in the WSNs by avoiding data transmission, whose cost could be prohibitively high for a continuous data gathering application.

Our approach to tackle the event-detection problem is by leveraging on both an architectural design framework and an algorithmic approach. To achieve distributed inference, we utilize a distributed compressed sensing model and data prediction, together with a threshold method as used in [5] to aggressively gather data when necessary. Our method could easily be applicable to a more sophisticated detection system. For example, the threshold method could be replaced with a learning technique such as in [6] or replaced by a sample splitting method [7]. For the remainder of this article, we will focus our attention on the *threshold method*, which could be applied to monitoring applications such as micro changes in climate condition, flood detection, wildfire detection, or applications where a sensor can detect a critical boundary of the measured value. Instead we leave the design of distributive inference solution to complex event detection problems such as face, pattern or speech detection, as an open research issue.

The contribution of this work is as follows: (1) We propose a data acquisition scheme for an event-based reactive network, which combines compressive sensing (CS) and prediction algorithms; (2) We design an adaptive sampling technique with feedback from the monitored event; (3) We validate the performance of SWIFTNET on a commercial off-the-shelf node; (4) We quantify the performance of SWIFTNET using a real test-bed environment to demonstrate that it is effective in prolonging the network lifetime of WSN in a wildfire monitoring application, despite being extremely light-weight.

Despite previous attempts to find solutions to data acquisition in WSN, to the best of our knowledge, none has a solution that combines compressed sensing and prediction algorithms. In this article, we have provided a comprehensive analysis of our design strategies, and we have shown how this mix can achieve a prolonged network lifetime using a real test-bed environment. It is our hope that this work would be beneficial to WSN developers and practitioners in the field.

The remainder of this article is organized as follows: In Section 2, we present a brief overview of previous work on monitoring application using WSN. In Section 2.1 we present the problem domain and the key questions tackled in this work, which is followed by the presentation of our solution and the design of our approach to the particular class of problems in Section 3. The use-case application scenario is presented in Section 4. We discuss the real test-bed environment and the presentation of results in Section 5. Finally, in Section 6 we conclude by summarizing and highlighting open research issues.

## 2. Background

Previous works have proposed the use of wireless sensor networks as a solution to various monitoring application scenarios. For example, [8] proposed FireNet, a WSN architecture for fire rescue applications. The work addresses some requirements and challenges for wildfire application. Similarly, in [9], the authors described a framework that included the system architecture, hardware, and software designs. Although this work provides insight on the necessary requirements for wildfire monitoring, it did not provide the framework implementation.

In addition, [5] proposed a design for wildfire monitoring using wireless sensor networks. The authors provided some field testings prescribed as test burns. One of the design goals is to investigate how a sensor node copes with high degree temperatures, without losing much of the sensed data. In another related literature [10], they proposed FireWxNet, a multi-tiered portable wireless system for monitoring weather conditions in a wild-land fire. All these works focus on framework and deployment architectures of WSN. They do not address the data acquisition protocol that runs on the sensor node devices and on the sink node.

Due to the energy spent in receiving and forwarding data, advances in hardware and software solutions have been proposed recently. From the hardware point of view, energy neutral designs are on the increase in the development of WSNs, such as in [11,12], while from the software point of view, an application-specific approach is proposed in [13,14]. Their method combines adaptive sampling and an energy-aware routing strategy for a flood warning system called FloodNet. One key elements in these studies is that, hybrid approaches have shown to improve the longevity of WSN deployment if properly designed.

A survey on energy conservation in WSNs was carried out by [15]. The study highlighted various design methods and the state-of-the-art techniques employed in this domain. Similarly, a more recent study by [16] used an adaptive sampling technique for a snow monitoring application. Their work revealed improvements on energy consumption compared with a fixed-rate sampling method. However, their approach is computationally intensive. In addition, the algorithm is executed at the sink for each sensor node, hence centralized. Sending frequent updates to several nodes in a lossy medium could be counter-productive. Moreover, their work focused on how to reduce oversampling in their application scenario and in some instances the nodes are switched off. This is different from our work, where the nodes are autonomous and where the goal is to achieve a continuous sampling, while limiting unnecessary communication without jeopardizing useful events in the monitored environment.

In a nutshell, we focus on the implementation of the software architecture that runs on the sensor nodes and the sink particularly designed to meet the requirements of a fast-reactive network. Our algorithm design can also be useful for any application that requires high responsiveness and a low data transmission, without necessarily missing any useful event throughout the monitoring operations. Furthermore, our approach can be implemented together with any of the frameworks described in [8,9]. For the purpose of empirical verification and due to straightforward method of testing our method, we present a wildfire monitoring application as a use-case. We believe our design approach can easily be extended to other similar event-based application scenarios.

### 2.1. Other Existing Methods

The research in WSNs in the past years have focused on two main areas namely: (1) Network related issues such as, routing and MAC layer designs and (2) Application related issues. In this article we are concerned about the application related issues, specifically we tackle the problem of distributed inference as it relates to in-network data suppression and communication reduction in WSNs. In the literature, learning models have been used to tackle this class of problem. Theoretically, models allow the possibility of examining fundamental questions of inference through learning techniques under tight communication constraints in wireless sensor networks. Some of these approaches have been well studied using parametric models, with the assumption that data to build a suitable model is available, and the system designers have prior domain knowledge of the application setting. However, if few data is available and there is limited application-specific knowledge, the non-parametric model building is preferred. From this stand point, the focus of this article is applying a combination of well-known alternative non-parametric learning-theoretical models, where data is sparse and prior knowledge of the environment is limited. Some of these models, such as compressed sensing, least mean square estimation, and regression estimation models have been widely studied in the field of signal processing, machine learning and in statistics.

One of the key research questions that arises in the literature is: are we able to apply these set tools for intelligent inference and data acquisition in WSNs? As discussed in [17], the classical limits of the algorithms for non-parametric learning are not always applicable in WSNs, in part because the classical models from which they are derived have abstracted away from the communication involved in the data acquisition process. We believe that this question can be addressed by understanding the fundamental limits learning models impose on energy and bandwidth. Thus we consider a solution that combines signal processing, data suppression and communication to the above problem.

In the next sections of this article, we briefly cover some key elements as it relates to our proposed design framework. Our intention is to jointly explore these key areas in order to achieve a robust WSN deployment.

#### 2.1.1. Data Compression

Data compression often studied in the field of machine learning and artificial intelligence has been widely used to tackle various application-specific problems. This method involves encoding information by using fewer bits than the original encoding. Compression is useful because it reduces resource usage. When applied to WSNs, it helps to reduce storage space and the burden on transmission capacity, which is vital for the network lifetime. However, because compressed data must be decompressed in order to use it, this extra processing imposes a computational cost, which is not easily offset. In WSNs it even becomes difficult to practically implement a data compression scheme due to the limited memory space and the battery capacity of the nodes. Thus requiring a space-time complexity tradeoffs to accomplish this in real-time.

In recent literature, a new alternative to the traditional data compression known as the Compressed Sensing (CS) has been proposed [18,19]. This new method offers a promising solution for data compression. Our focus in this article is to show the necessary tradeoffs that can be accomplished when this new method is used for intelligent data acquisition in wireless sensor networks.

For data gathering application, CS is usually adopted to increase the overall networking efficiency [20,21]. In this case, CS aggregates the data during the collection in a multi-hop network, thus avoiding a progressive increase of the payload that is being transmitted from the peripheral nodes to the sink [22]. The work in [23] introduces an approach to minimize the network energy consumption through joint routing and compressed aggregation. Differently from our approach, there is no adaptive mechanism to activate the CS. The method provides an optimal solution which is Nondeterministic-Polynomial (NP)-complete, hence the authors present a greedy heuristic that delivers near-optimal solutions for large-scale WSNs. Similarly, the approach presented in [24] uses the results from studies in vehicle routing. The protocol is applied to data collection in WSN and integrated with CS. Unfortunately, even this approach is NP-hard, and the authors define both a centralized and a distributed heuristic to reduce the computational effort. These works analyze the routing and aggregation problem in WSNs, while in our work, we focus on the adaptive strategies when the networks is required to operate at different rates; since it allows the sensor nodes to have duty-cycle activity and hence reducing the overall payload transmitted in the network.

In recent literature [25] proposed the use of compressed sensing with a scheduling approach. They used compressed sensing with measurement scheduling and estimation on in-situ soil moisture sensing. In addition, [26] proposed a Non-uniform Compressed Sensing (NCS) method that exploits compressibility and heterogeneity for an improved performance. Their approach is compared with the traditional CS using a rain-forest application that monitors wind speed and direction. Although, these works addressed the problem of how to apply CS to soil moisture processes and NCS to rain forest restoration processes in a non-real-time fashion, they both differ from our work. These works focused on the accuracy of the sensing phenomenon, while tuning the selection of the measurement matrix and representation basis to conform to the application domain. Instead, our work tackles the joint problem of energy consumption and accuracy by using a combination of CS and data prediction.

In another related work [27], the authors propose an adaptive data gathering scheme based on CS to address the problem of reconstruction quality degradation due to sensed data variation. They incorporate the Auto-regressive (AR) model to exploit the local spatial correlation between sensed data of neighboring sensor nodes, the reconstruction is thus adaptive to the variation of sensed data by adjusting the AR parameters. This work solely tackles the problem of reconstruction quality and not the over-aching energy consumption problem in WSN. In addition, in [28], the authors propose a method for data acquisition using the hierarchical routing method and compressive sensing for WSN, similar to the work in [24], instead their work is targeted at verifying suitable methods for a large region covered by a multi-hop network.

The work in [29] proposes a data acquisition method for accurate signal estimation in wireless sensor networks. Their method uses a concept of “virtual clusters”, to form groups of sensor nodes with the same spatial and temporal properties. Two algorithms are used, namely: The “distributed formation” algorithm, which automatically forms and classifies the virtual clusters and the “round robin sample scheme”, which schedules the virtual clusters to sample the event signals in turn. Although the authors propose a data acquisition scheme using a combination of round-robin and virtual clusters, this is different from our approach where we propose a combination of CS and data prediction to achieve real-time data gathering at low-cost.

Other literature exists [30,31] wherein they propose hybrid methods that combine CS and other approaches notably routing, none of these works propose to use CS and data prediction scheme. Moreover, the focus of these previous studies is centered on how to achieve better reconstruction with respect to the sparsity of the signals and the recovery methods. Indeed, given any averagely sparse signal with reconstruction matrices, we are interested in designing a real-time data acquisition scheme that allows fast-detection of interesting events and to simultaneously achieve low energy consumption in light of energy intensive tasks. This requires each node to sample data autonomously depending on the rate of change of the signal, while keeping energy consumption low in the entire network. Consequently, to achieve this goal, our method uses a combination of CS, prediction, and adaptive sampling strategies. And as a further proof of concept, we have demonstrated our work using a real-world testbed implementation.

It is worthy of note that while the design of CS with routing algorithm could also improve longevity of WSN deployments, we have not investigated this approach, instead we envision that the combination of our method with any of the proposed routing algorithms described in previous works could yield more attractive results for WSN deployments. The design of this approach is currently out of scope and is therefore left as an open research issue.

#### 2.1.2. Data Prediction

Data prediction on the other hand, has received lots of attention in various application domain in WSNs, due to the ability to use this simple and yet powerful technique to suppress data in the network. Part of this method involves training models that could be used to predict future data traces, both at the base station and on the nodes. One of the areas in which this method is useful is in environmental monitoring application, where the goal is to follow both space and time evolution of the physical phenomena. Most of the prediction models often considered in the literature aim at approximating or predicting sensor measurement, which generally takes the form

(1) $$M_{\theta }:ϒ\to \mathbb{R}$$

(2) $$x\mapsto {\hat{n}}_{i}\left[t\right]=M_{\theta }\left(x\right)$$

where *n̂_i_*[*t*] denotes the approximation of the value of a sensor node *n_i_*[*t*] at time *t*, and *x* is the sensor measurement, which could be temperature, wind speed, humidity, *etc*. The real task of this method is how to build a model *M* such that a bounded approximation error *ϵ* is minimized *i.e.*,

(3) $$\left|n_{i}\left[t\right]-{\hat{n}}_{i}\left[t\right]\right|<\epsilon ,\forall i\in N,t\in T$$

Various optimization techniques [32-40] have been employed in literature to tackle the choice of the model buildup. Arguably these methods have yielded improved resource management with reduced communication cost in WSNs. One of our focus is how to apply this data prediction approach, together with a data compression method introduced previously to achieve a further improvement in communication reduction.

In the next section, we discuss our solution to event-detection problems, which is applicable to the *threshold method* as mentioned earlier. Furthermore, we examine a use-case scenario where our design model is applicable.

## 3. SWIFTNET: Protocol for Fast-Reactive Monitoring

SWIFTNET is an algorithm that combines the two well-known methods described above namely; a compressive sensing algorithm and a prediction algorithm together with an adaptive sampling strategy, which is designed to achieve fast-reactiveness and a prolonged network lifetime for WSNs. To meet the needs for an adaptive sampling strategy, we deviate from the traditional prediction approach with fixed sampling interval. Rather we allow the prediction algorithm to adjust the sampling interval depending on the incoming sensed data. With respect to event-detection applications, SWIFTNET is able to react quickly to varying changes in the measured values, limit the unnecessary data transmission, and yet provide accurate reconstruction of signals.

**Figure 1:**
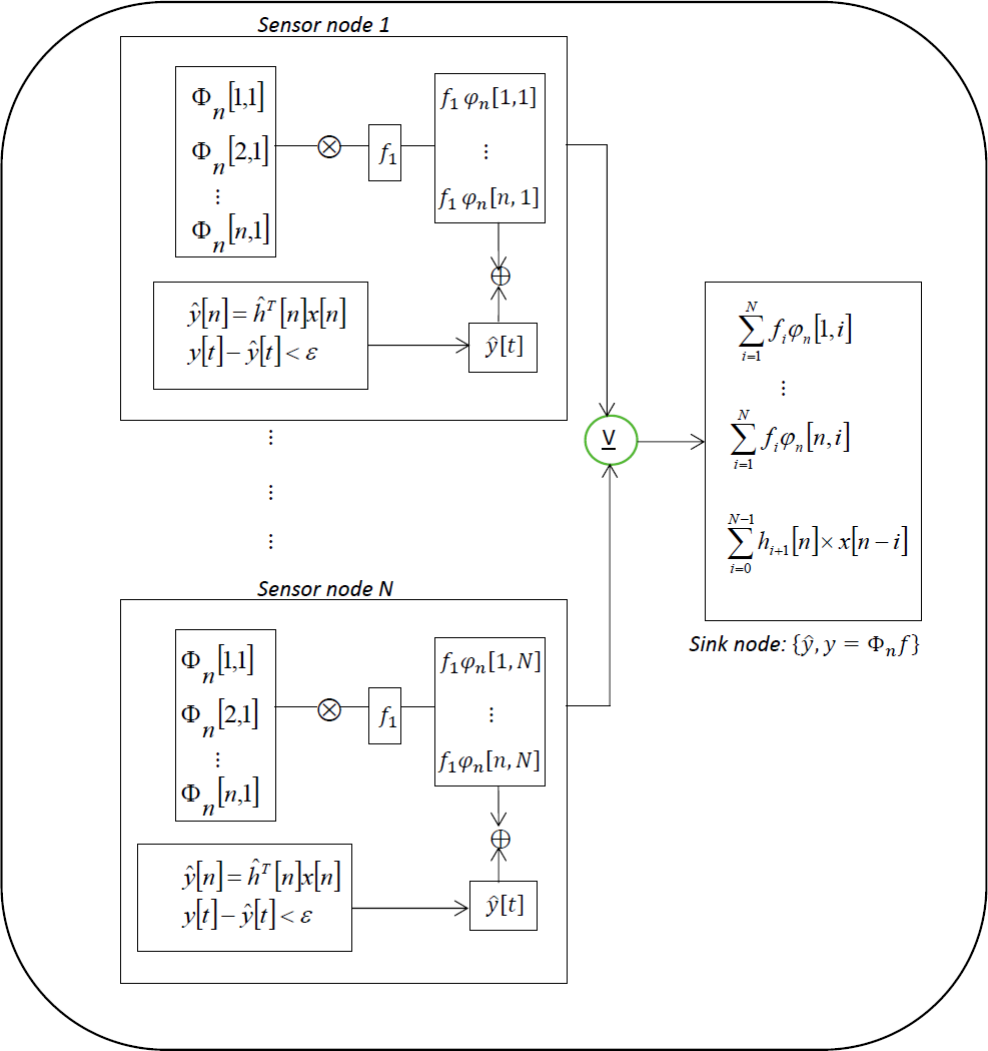
SWIFTNET in a WSN with star topology.

In Figure 1, we show a schematic diagram of SWIFTNET in a WSN with star topology. The figure reveals how each node implement the SWIFTNET algorithm. For example, *Sensor node 1* compresses the data traces and if necessary performs data prediction. The resulting vector is then transmitted to the *Sink node* for decompression and processing. However, the key task in each node is how to simultaneously implement the data compression and prediction algorithms, while aggressively acquiring measurement samples in real-time. In the next section, we discuss in detail the theoretical features that underlie the SWIFTNET design solution, *i.e.*, compressed sensing, data prediction, and adaptive sampling.

### 3.1. Compressed Sensing

The basic theory of CS emerged in the works of [18,19]. CS is a simple and yet efficient data acquisition paradigm. Precisely, CS exploits the fact that many natural signals are sparse in the sense that they can be compressed as long as they can be represented in their proper basis Ψ. CS is governed by two principles; sparsity and incoherence.

Sparsity relates to the fact that the information rate of a continuous signal may be smaller than its bandwidth. On the other hand incoherency, as explained in [41], extends to the duality between time and interval, and therefore it expresses the idea that objects having a sparse representation in Ψ must be spread out in the domain in which they are acquired. With this knowledge, it is possible to design an algorithm or sensing protocol that captures useful events that are contained in a sparse signal, and then compress it into a smaller amount of data before transmission. It is on the basis of these premises that we argue to use CS in combination with other strategies as a data acquisition scheme for a surveillance application. Let us consider a discrete-time signal x, viewed as a column *N* × 1 vector in a ℝ^n^ domain. The signal can be represented in terms of a basis of *N* × 1 vectors $\psi_{i=1}^{N}$. For simplicity sparsity offers that many natural signals can have a concise representation when expressed in a convenient basis. Mathematically, the vector *x* ∈ ℝ*^n^* can be expanded in an orthonormal basis such as Discrete Cosine Transform (DCT) or wavelet given as:

(4) $$x\left(t\right)=\sum_{i=1}^{N}y_{i}\Psi_{i}\left(t\right)$$

where *y* is a coefficient of *x* in the domain *y_i_* = [*x*, Ψ*_i_*]. Similarly, Ψ can be expressed as an *N* × *N* matrix with [Ψ_1_Ψ_2_…Ψ*_N_*] as columns. The implication is that when a signal has a sparse expansion, one can discard the small coefficients without much perceptual loss [18]. Generally, any basis vector Ψ can be formed without assuming prior knowledge of the signal, apart from the size, since the size determines the size of the measurement matrix Φ.

The theory of CS demonstrates that a signal can be compressed using a proper measurement matrix Φ = [*φ*_1_, *φ*_2_, …, *φ_N_*] of size *M* × *N*, with *M* < *N*. Having said this, we are interested in *under-sampled* situations, where the number of measurements *M* available are much smaller than the dimension *N* of a signal *y*. Assuming *y* is the compressed form of *f*, then the matrix form is given as:

(5) y=Φf

where *y* is an *M* × 1 column vector. The recovery process of the original signal *f* can be achieved through optimization methods. According to [41], suppose a given pair of orthobases of ℝ*^n^* given as (Φ, Ψ), where Φ is used for the sensing phenomenon and Ψ is used to represent *x*, then the coherence between the sensing basis Φ and the representation basis Ψ can be expressed as:

(6) $$\mu \left(\Phi ,\psi \right)=\sqrt{N}\cdot \max_{1\leq k\leq N}|\langle \varphi_{k},\psi_{j}|\rangle$$

If Φ and Ψ contain correlated elements, we say the coherence is large. It follows that the smaller the coherency, the better the reconstruction would be. From linear algebra, the upper and lower limit is bounded as $\mu \left(\Phi ,\Psi \right)\in \left[1,\sqrt{n}\right]$ [41]. This relationship form the basis for which we are able to recover sparse signals.

#### 3.1.1. Signal Recovery in Sparse Domain

In general there are the following classes of sparse signal recovering methods. The first class solves *b̃* with the smallest *l*_0_ norm:

(7) $$\min_{\tilde{b}\in {\mathbb{R}}^{n}}{\left\|\tilde{b}\right\|}_{l_{0}}\text{subject to}y_{k}=\Phi \Psi \tilde{b}$$

Solving Equation (7) above is intractable [42]. There are other faster algorithms designed to solve this set of problem e.g., using the Smoothed *l*_0_ (SL0) norm method proposed by [43]. Their experimental results revealed that the proposed algorithm is about two to three orders of magnitude faster than the state-of-the-art interior-point Linear Programming (LP) solvers such as the Basis Pursuit (BP) [44,45].

The second class seeks to solve the *l*_1_-minimization problem (also known as the Basis Pursuit problem) through linear programming. For example, using this second class approach, the result from [18] asserts that, if *x* is sufficiently sparse, recovery via *l*_1_-minimization is possible. Normally, we would like to sample all the *n* coefficients of *x*, however, we can only get to sample a subset of these, such that, we can recover the data through Equation (8) given below:

(8) $$y_{k}=\langle x,\psi_{k}\rangle ,k\in M$$

where *M* ⊂ {1,…, *N*} is a cardinality constraint *M* < *N*. Thus, the reconstruction of *x* is either through a greedy algorithm, as proposed in [46], or using *l*_1_-norm minimization. The proposed reconstruction of *x* through *l*_1_-norm minimization is given by x* = Ψb*, where b* is the solution to the convex optimization problem (‖*b*‖_l1_, := ∑*_i_* |*b_i_*|) [41]:

(9) $$\min_{\tilde{b}\in {\mathbb{R}}^{n}}{\left\|\tilde{b}\right\|}_{l_{1}}\text{subject to}y_{k}=\langle \psi_{k},\Psi \tilde{b}\rangle ,\forall k\in M$$

From Equation (9), minimizing *l*_1_ subject to linear equality constraints can be solved using LP. This leads to the following *Theorem 1*, see proof in [47]:

Fix *x* **∈** ℝ*^n^* and suppose that the coefficient sequence *b* of *x* in the basis Ψ is *K-sparse*. Select *m* measurements in the Φ domain uniformly at random. Then if,

(10) $$m\geq C\cdot \mu^{2}\left(\Phi ,\Psi \right)\cdot K\cdot \log n$$

for some positive constant *C*, the solution to Equation (10) is exact with overwhelming probability [41], see proof of *Theorem 1* in [47]. With the concept of sparsity and incoherency in mind, the problem of CS is therefore reduced to: (1) finding a stable measurement matrix Φ such that the important information embedded in any *K*-sparse signal can be recovered easily and (2) finding an algorithm that can recover the *x* signal in Equation (4) from any *m* ≈ *K* measurements. It follows that:

No significant information is lost by measuring any set of *m* coefficients that is far less than the signal size. If *μ*(Φ, *Ψ)* is close or equal to one, the order of *K* log *n* samples is enough.
The role of incoherency becomes transparent *i.e.*, the smaller the coherency, the fewer the samples that will be needed for reconstruction.
We just run the algorithm; if the signal is well sparse, exact reconstruction is possible, *i.e.*, the signal *x* is exactly recoverable from set *m* by minimizing a convex function, without assuming knowledge of the non-zero coordinates of *b*, the amplitudes or locations, which are unknown *a priori*. In real-world applications, the signal will be invariably corrupted by some level of noise; however, a small perturbation in the signal should cause small perturbation in the recovery process.

From the above signal recovery analysis, the task of finding and implementing a stable measurement matrix on a 32-bit node platform such as the WISPES W24TH is non-trivial, since the nodes are limited in memory, and the requirement of most event-detection applications necessitates a real-time update of the events, hence a real-time signal recovery method. In light of these limitations posed by the application and the hardware unit, the key question is whether it is still possible to exploit CS to a real-time data recovery application.

Next, we examine the components of the CS sensing matrix, and the conditions that are required for a sparse signal reconstruction.

#### 3.1.2. Sensing Matrix

Given a sensing matrix *M* = ΦΨ, where Φ is an *m* × *n* measurement matrix drawn randomly from suitable distribution and Ψ is an arbitrary orthobasis, it has been shown by [41] that, to have a robust signal recovery, the sensing matrices must obey the Restricted Isometric Property (RIP) condition, *i.e.*, matrices with the property that column vectors taken from arbitrary subsets are nearly orthogonal. Thus, the larger these subsets, the better the recovery process. According to [41], it has been proven that any random matrix generation that obeys the RIP condition [42] is suitable for a signal recovery process, provided that

(11) m≥C⋅Klogn(n/K)

where *C* is a constant chosen depending on the instance. Consider the following matrix generation processes:

1. Generate matrix *M* by sampling *n* column vectors uniformly at random on the unit sphere of ℝ*^m^*.
2. Generate matrix *M* by sampling independent identically distributed (i.i.d) entries from a normal distribution with variance = *1/m* and mean = 0.
3. Generate matrix *M* by a random projection *P* as in “Incoherent Sampling” and normalize: $M=\sqrt{n/m}P$.
4. Generate matrix *M* by sampling independent identically distributed entries from a symmetric Bernoulli distribution $\left(\text{P}\left(M_{i,j}=\pm 1/\sqrt{m}\right)=1/2\right)$.

For all these example matrices, the probability of sampling a matrix not obeying RIP when Equation (11) holds is exponentially small in *m* [41]. The authors further asserted that using a randomized matrix, together with *l*_1_ minimization, can offer a near-optimal sensing strategy. Alternatively, if we fix the orthobasis Ψ and generate a measurement matrix Φ according to any of the rules in Conditions (1)–(4) above, then with an overwhelming probability the matrix *M* obeys the RIP constraint, provided condition (11) is met [41]. This means, we can accurately reconstruct nearly sparse signals from under-sampled data in an incoherent domain. We therefore anchor the design of SWIFTNET on these assertions, by generating matrix *M* and sampling *n* column vectors uniformly at random on the unit sphere of ℝ*^m^* with the hope that we can solve the convex optimization problem using the *l*_1_ minimization method (see Equation (9)). One key contribution of our approach is that we have been able to successfully apply practically these theories to achieve an energy-efficient data acquisition method that is more robust than the traditional sense-transmit or prediction approaches.

Next, we present the prediction algorithm we have used in our design.

### 3.2. Learning Technique: Normalized Least-Mean Square Filter

In order to realize our goal of an aggressive data gathering process with minimal data transmission, we anchor our design on a light-weight learning method. The aim is to apply this model as a predictor in a distributed manner on both the sensor nodes and the base station. The Normalized Least-Mean Square (NLMS) filter is known to be an effective learning model, which could yield optimal results. NLMS is a class of adaptive filters that are well-known as an alternative solution to perform prediction on time series data without requiring knowledge of the statistical properties of the phenomenon being monitored. The algorithm has three parts as discussed in [48] namely:

1. The training sample: this includes the input signal vector denoted as *x*(*n*) and the desired response denoted as *d*(*n*).
2. Adaptive parameter: this includes the learning rate denoted as the *η* and the weight initialization *h*(*x00302*), which is set as *h*(*x00302*)(*0*) = 0.
3. The computation: this includes the computation of the error signal, the weight adaptation and the prediction.
  The error signal is calculated as:
  (12) $$e\left[n\right]=d\left[n\right]-{\hat{h}}^{T}\left[n\right]x\left[n\right]$$
  The weight vector is then updated as:
  (13) $$\hat{h}\left(n+1\right)=\hat{h}\left(n\right)+\frac{\eta x\left(n\right)e\left(n\right)}{{\left\|x\left(n\right)\right\|}^{2}}$$

**Figure 2:**
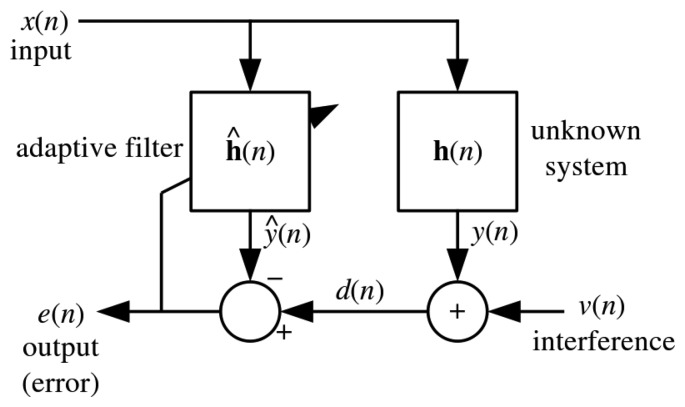
LMS Filter.

The NLMS [49] is a variant of the stochastic gradient descent LMS filter shown in Figure 2. The difference is that the NLMS offers guaranteed convergence by normalizing the filter with the power of the input as shown in Equation (13). Basically, an adaptive filter takes in a sample *x*[*n*] of input signal *x* at each step *n* to compute the filter output *y*[*n*] given as:

(14) $$y\left[n\right]=\sum_{i=0}^{N-1}{\hat{h}}_{i+1}\left[n\right]\times x\left[n-i\right]$$

A prediction algorithm can be constructed with a linear combination of the past *N* samples of the input signals with the weight adaptation *h_i_*[*n*] from the filter. The output signal *y*[*n*] is then compared with the desired signal *d*[*n*] which the filter tries to adapt to. These weights are computed to satisfy the optimality criterion which is normally the minimization of the Mean-Squared-Error (MSE). The weights are updated at each *n* step with the aim to iteratively converge at the minimum. The error signal e [*n*] is used in the adaptation rule in Equation (13) in order to reduce the errors in the subsequent step *n* + 1.

Using the prediction algorithm does not meet our need for an adaptive prediction. Since a prediction model uses a fixed sampling interval, we are interested in following the evolution of the signal at a faster rate when necessary. Thus, to satisfy this need, we integrate an adaptive sampling strategy into the prediction model, which we discuss next.

#### 3.2.1. Adaptive Sampling Strategy

We design an adaptive sampling mechanism that takes inputs from the monitored environment with units in seconds. We express the sampling interval γ in the form:

(15) $$\gamma =\beta \cdot \theta_{\min\left(t\right)}/X_{\text{sense}}$$

where *θ_min|max_*_(_*_t_*_)_ > 0 denotes the minimum or maximum non-negative value that could be attained within a specified sampling period. For example *θ* is *min* when our intention is to capture high values, and conversely, *θ* is *max* if the interest is to capture low values in the monitored environment. From Equation (15) *β* denotes the initial sampling interval specified for the WSN deployment. *X_sense_* is the sensed parameter e.g., temperature, humidity, wind speed at time *t*. Both *θ_min_*_(_*_t_*_)_ and *β* values can be set by the user based on the application-specific knowledge.

Suppose we consider a wildfire monitoring application as a use-case, where our interest is to monitor high temperature values, *θ* is chosen as *min* e.g., *θ_min_*_(_*_t_*_)_ could be set to 2 °C (assuming it is the lowest non-negative and non-zero temperature value recorded for the sampling period). Obviously the lower this value is, the smaller the sampling interval γ, hence the higher the sampling rate. Due to the inverse relation between γ and *X_sense_* from Equation (15), an increasing air temperature automatically increases the sampling interval and vice versa. For each data transmitted during the prediction phase, the time stamp is added to the data packet with the newly computed sampling interval. With this design, the prediction algorithm is adaptive and highly responsive to changes in the air temperature signal. The same metric is applicable to other parameters such as humidity, wind speed, *etc*. For the remainder of this article, we shall refer to the integration of the adaptive sampling strategy and prediction model (Section 3.2) as an “Adaptive prediction” method.

### 3.3. Our Design of Measurement and Representation Basis

In this section, our discussion covers the selection of the measurement matrix $ on the W24TH node platform. The measurement matrix corresponds to the basis for which matrix *y* corresponds to the compressed form of a signal *f*, whereas the representation basis Ψ is used in the reconstruction algorithm corresponding to the sparsity of the original signal *f* during the recovery process.

#### 3.3.1. Measurement Matrix and Sparse Representation

Recall from Section 3.1, that one of the aims is to find a sensing matrix with the property that column vectors taken from arbitrary subsets are nearly orthogonal. Hence, the larger these subsets are, the better will be the reconstruction. In general, random matrices are largely incoherent with any fixed basis Ψ. For example, it is proven by [41] that if we select an orthobasis $ uniformly at random, which can be done by orthonormalizing *n* vectors sampled independently and uniformly on the unit sphere, then with a high probability, the coherence between Φ and Ψ is about $\sqrt{2\log n}$. To achieve this, we used the Linear Feedback Shift Register popularly known as LFSR in the field of electronics.

**Figure 3:**
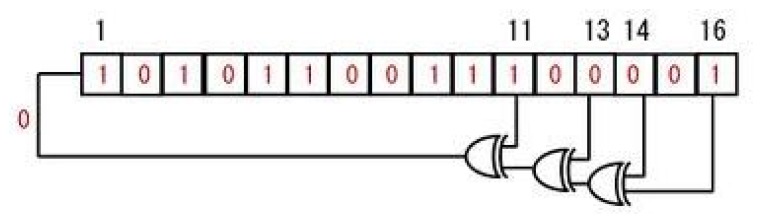
A 16-Bit LFSR.

An LFSR is a shift register that is capable of generating pseudo-random numbers, which when clocked advances the signal through the register from one bit to the next most significant bit. Linear feedback shift registers make extremely good pseudo-random pattern generators (PRNG). An LFSR can be formed by performing an exclusive-or (XOR) on two or more of the flip-flop outputs, and feeding them back as inputs into one of the flip-flops shown in Figure 3. This figure shows a 16-bit LFSR, the bit positions that affects the next state are called taps. From this figure the taps are [16,14,13,11], the rightmost bit of the LFSR is the output bit. One way of implementing LFSRs is to have the taps XORed sequentially and then fed back into the leftmost bit. This way the XOR is external from the shift register. Another way of implementing the LFSR is by making the XOR internal to the register. In our implementation, we have utilized the external XOR method with a 16-bit feedback polynomial of maximal period 65,535 (*i.e.*, 2*^n^* − 1, with *n* = 16). The output is initially loaded with a seed value (in our case 0xACE1u), also known as the initial state, and when the register is clocked, it will generate a random pattern of 0 s and 1 s.

A previous study in [25] conducted a performance evaluation of the different measurement matrices for signal recovery using CS. Instead, our method addresses the practicability of using one of these matrices on a 32-bit architecture with minimal energy requirement. It implies that, given any measurement matrix with a proper representation basis, we are able to design a reactive algorithm that achieves a minimum energy consumption, without losing any interesting event in the monitored environment. One of the key design choices requires that the chosen matrix should be easily implemented on the W24TH node prototype without incurring huge computational overhead and memory usage.

As explained under the CS theory, recovering sparse signals requires random matrices. Recall from Section 3.1.2 that one of the potential methods that obeys the RIP condition is to generate a matrix of uniform distribution. The generation of these random matrices requires producing a sequence of random numbers and storing them into arrays of size *M* × *N*. The LFSR is a potential solution for this since uniform random numbers can be easily generated with low complexity, considering the limited memory capacity of the 32-bit WISPES W24TH node platform. In previous study by [50] (pp. 8-11), it was shown that it is possible to generate deterministic sensing matrices from an additive character sequence using an LFSR. Their results revealed that the sensing matrix generated using the LFSR has better eigenvalues statistics than the Gaussian random matrices, hence more suitable for CS matrices. Similarly, the study by [51] revealed that LFSR is suitable for generating uniform random numbers and they further showed some hardware implementations of the LFSR. In [52] (p. 4064) an LFSR was used to generate a Toeplitz matrix for a special-purpose light sensor application, for which the output is fed into an accumulator. The output is then sampled in a pseudo-random fashion, and it was shown to meet the requirement of a CS matrix. From these previous studies, and from our observation, an LFSR is sufficient for generating column vectors of a matrix that is sampled uniformly at random. Hence it meets our needs with respect to the CS matrices, as we shall see from our experiments, especially since our aim is only to perform compression on the streams of signals, and then reproduce the same matrix using any of the fixed basis Ψ such as the DCT at the sink node for the reconstruction. Various studies have investigated the selection of a proper basis Ψ for CS problems, for example the work in [53] used diffusion wavelets [54] as an alternative to the conventional DCT and Fourier compression bases, due to the goal of achieving a high fidelity data recovery. The choice of these methods is currently out of scope of this study. However, from our experiments DCT proves to be sufficient for the signal recovery process as we shall see in the next sections.

### 3.4. Signal Reconstruction Algorithm

Algorithm 1 shows our proposed signal reconstruction algorithm. We assume the sink to be sufficiently powerful e.g., a node directly connected to a laptop or to a high-end PC with enough computational resources. The sink node should be capable of recovering the compressed packets received stored in a vector *comp_vector_* shown in Line 2 of the algorithm. On Lines 4 to 11 the LFSR measurement matrix is generated. Following this the signal is reconstructed shown on Lines 13 to 16. The function *dctmtx* represents the DCT Ψ basis and the *SolveBP* function executes the linear programming (LP) code written in MATLAB obtained from Sparselab [55]. However, to be able to recover the signals, the sink node must have a knowledge of the sensing matrix used by the node for compression. To achieve this, the nodes must be able to generate such a matrix and then communicate it with the sink. Unfortunately, the leaf nodes have limited memory capabilities to store the matrix. Moreover, transmitting several columns of vectors will equally require the radio to be in “ON” mode, and this would be counter-productive to the goal of minimizing energy consumption in the network. A solution to the above problem will be to find a matrix that we can construct without storing it on the leaf nodes or transmitting it to the sink. Thus we generated the measurement matrix Φ by sampling *n* column vectors from a uniform distribution using the LFSR as described in the previous section. Consequently, the matrix can be generated on the leaf nodes with a “seed” and simultaneously we can perform data compression. This way it is not needed to store the generated matrix on the nodes, rather the LFSR matrix using the same “seed” can be utilized for the reconstruction process by applying the proper basis Ψ at the sink node.

In a nutshell, the compression phase is accomplished within the network using Equation (5). In contrast, the decompression of the signal is performed at the sink using Algorithm 1, as long as it has the knowledge of the measurement matrix Φ. With this method of matrix generation and simultaneously performing compression at the nodes, the memory usage is optimized, as we have been able to avoid storing the *n* × *n* matrix for transmission to the sink. In addition, the nodes are able to work autonomously.

An important observation from the experiments reveal that, if the signal is averagely sparse, the signals can be recovered with a high accuracy. From the above algorithm design, the solution to the problem of reconstruction requires *N* ≥ *cK* random projections, with *c*(*S*) = *O*(*log*(1/*S*)) and *S* = *K*/*N* denoting the sparsity rate of the signal. Obviously, the recovery error depends on the number of samples taken, and ideally, the error decreases for an increasing value of *N*. In [56], they provided a statistical estimation for when *K* is far less than *N;* they asserted that the data can be reconstructed with a high probability when c ≈ [3, 4], *i.e.*, taking *N* ≥ *3K* ≈ 4*K* random measurements should suffice. Thus, we set *K* to be 25% of the original signal.



**Algorithm 1** Reconstruction algorithm using LFSR measurement matrix.
1:lfsr ← 44257 #Initialize LFSR seed2:*comp*_(_*_vector_*_)_; *Sizeof Historytable*;3:#Create the measurement matrix by sampling *n* column LFSR vector:4:**for** (*i in* 1 : len(*comp*_(_*_vector_*_)_)) **do**5: **for** (*i in* 1 : *Sizeof Historytable*) **do**6:  #Generate the LFSR vector used for compression at the source nodes:7:  bit := ((lfsr ≫ 0) ˆ (lfsr ≫ 2) ˆ (lfsr ≫ 3) ˆ (lfsr ≫ 5)) & 1;8:  lfsr := (lfsr ≫ 1) (bit ≪ 15);9:  matrix(i,j) := lfsr;10: **end for**11:**end for**12:#Signal reconstruction using LFSR generated matrix:13:CompA := matrix × *comp*_(_*_vector_*_)_;14:*B_rec_* := dctmtx(matrix) × len(comp*_(vector)_*);15:CoefBP := SolveBP(matrix × *B′_rec_*, CompA, len(comp*_(vector)_*));16:Recon := (*B′_rec_* × CoefBP);


To complete the SWIFTNET design, the adaptive prediction algorithm discussed in Section 3.2.1 is implemented on top of the CS. In addition, it is invoked when a user defined threshold *ρ* is reached. This value can be chosen by the user depending on the application need. Similarly, as previously discussed, other learning-based methods can be applied when choosing this threshold function. In addition, depending on the scenario, any prediction model could suffice. However, this choice should be well guided, and we defer this discussion to a later section.

## 4. Use Case: Application Scenario

The applicability of SWIFTNET design strategy is vast e.g., it can be applied to any event detection application such as, seismic monitoring [57], soil moisture measurement for rainfall detection [25], wildfire monitoring [58], flood warning system [13], *etc*. For easy testing and for the implementation of our approach, we present a wildfire monitoring application as a use-case.

### 4.1. Wildfire Characteristics

Often in a forest reserve or wild-land, there is frequent surveillance from towers, ground, and aerial patrols for wildfire. Sometimes, there is a fleet of airplanes regularly patrolling over the forest looking out for wildfires or for any abrupt changes in the environmental conditions that could trigger a fire event at various time intervals of the day or week, especially in fire-prone regions such as in Australia, Greece, Alabama, *etc*. In addition, the public can often report wildfires 24 h a day through a toll-free telephone system. When a fire is reported, a dispatch center sends fire-fighters and volunteer fire departments as needed to suppress it.

In this instance, there are normally two stages: (1) a fire alarm is triggered either manually or automatically and (2) a rescue operation is initiated to suppress it. In the former case, environmental variables such as air temperature, the wind speed, humidity and atmospheric pressure are monitored. One of the most monitored characteristics is the air temperature, often of particular interest is the range between 40 ^°^C to 46 ^°^C, termed the “emergency zone”. In this zone, there is the possibility for the alarm to be set-off and/or a trigger of immediate observation of the environment.

In the latter stage, assuming an alarm is set-off, combating the situation is non-trivial, especially in a case of “series of bushfires”; this could continue for days before it is eventually suppressed completely. At this stage lots of efforts are put in place, the ability to combat the bushfire will increase the number of lives and amount of property that can be salvaged including the lives of the fire-fighters. For example, in some circumstances where several homes are threatened by a wildfire, the Forestry Commission can call-in helicopters with large water buckets. These buckets do not put out the fire, but reduce its intensity so that the fire-fighters can more easily suppress it. The helicopter service is extremely expensive and it is only employed in severe fire conditions. An early warning system that can provide continuous updates, with an accurate view of the incident as the fire-fighters battle on, is very crucial to the successful operation of combating the fire incident.

The Alabama Forestry Commission [59] argued that an integrated approach of multiple systems can be used to merge satellite data, aerial imagery, and personnel position via a Global Positioning System (GPS) into a collective whole for near real-time wireless interaction with the Incident Command Centers (ICC). We believe that the use of WSNs together with these systems is crucial, as they are useful in providing localized and ground information to the other systems. A complete solution including an early warning system that reports a wildfire right from the on-set is, crucial. The use of wireless sensor networks could play a very important role in such an operation.

We devote the next section to describe how the SWIFTNET design framework can be applied to a wildfire monitoring application, followed by the discussion of a real test-bed implementation.

### 4.2. SWIFTNET Design Illustration for a Wildfire Application

**Figure 4:**
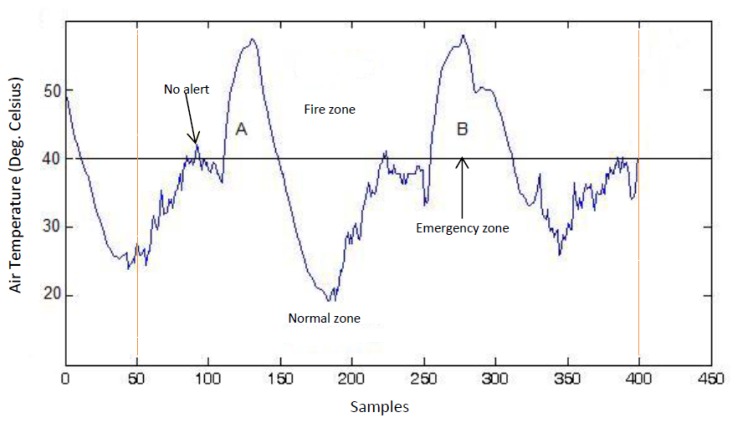
SWIFTNET: design illustration.

Figure 4 illustrates SWIFTNET design strategy. The points of interest between 50 to 400 sample points are shown with the dotted lines. This figure shows an air temperature data trace from a wildfire monitoring application. To illustrate, the diagram is partitioned into two zones of interest:

1. **The Normal Zone**: In this zone, since the temperature is below the threat level or potential fire hazard, a low sampling interval would suffice. In SWIFTNET, this zone is controlled by the CS algorithm.
2. **The Fire Zone**: In this zone, locations A and B are the major points of interest in the event of a combat operation during a wildfire disaster. A higher sampling interval and an aggressive data collection would be necessary, the more information gathered during these periods, the better the operation is likely to be. Similarly, the longer the network lifetime achieved during the fire incident, the better the inference that would be generated. Consequently, we strive for high quality information reporting and longer lifetime deployment. To achieve our goal in SWIFTNET, this zone is automatically controlled by our adaptive prediction algorithm.

From Figure 4, the emergency threshold *ρ* is introduced to dynamically switch between CS and the adaptive prediction algorithm. This threshold temperature can be set by the users, depending on the past history of wildfire events, or it could be learned from the historic data if necessary. When the air temperature is below *ρ, i.e.*, in the Normal zone, the CS algorithm is invoked in SWIFTNET, which samples the environment at a fixed sample interval *β*. In our experiments, we set *β* to 5 min, which amounts to 288 samples in a 24-hour period. The sparsity *K* is set to 25%, which is 72 non-zero samples collected and transmitted in one packet every 24-hour period.

Similarly, when the algorithm switches to prediction, the prediction phase uses a user defined error bound ϵ*_max_*, which we set to ±0.3 °C *i.e.*, <1% error margin. The error bound controls whether data is transmitted or suppressed. In addition, it contributes to the rate of reactivity of the network.

Recall from Equation (15) that an increasing *X_sense_* automatically increases the sampling interval, and vice versa. With this design, the prediction algorithm is adaptive and highly responsive to changes in the air temperature signal. Similarly, in order to prevent false alarms, SWIFTNET uses a buffer window size *n* in the prediction model. As mentioned previously, once the threshold *ρ* is reached, and if the user error bound ϵ*_max_* condition is met within the computed γ sampling interval, an alarm is automatically invoked and data transmission resumes. However, if this condition is not met, the prediction phase does not start at the sensor nodes until the window size *n* is full, which is then transmitted to the sink node without invoking a fire alarm. For example, in Figure 4 we note the few spikes between 90 to 100 sample points, where the temperature rises above the *ρ* value, but quickly drops below the emergency zone, hence, no alarm is triggered due to the reasons aforementioned. Apart from the reactivity of the network being controlled by *X_sense_*, decreasing the user error bound equally increases the reactiveness of the network and vice versa. The additional advantage of SWIFTNET is that, it requires no prior knowledge of the monitored environment. The algorithm is light-weight and can be practically implemented on any existing 32-bit node platform that is currently available in the market.

In summary, since the majority of the time the air temperature could be significantly lower than the threat level, by using CS for the data gathering during this period, significant savings on energy resources are achieved without a significant loss in accuracy in the measured values. Sensing will continue at a low sample interval, as long as the signal is below the emergency threshold. On the other hand, if the air temperature increases above the threat level, we can detect any event of interest by using the adaptive prediction algorithm, which also amounts to additional energy savings compared with a traditional sense-and-transmit method.

**Figure 5:**
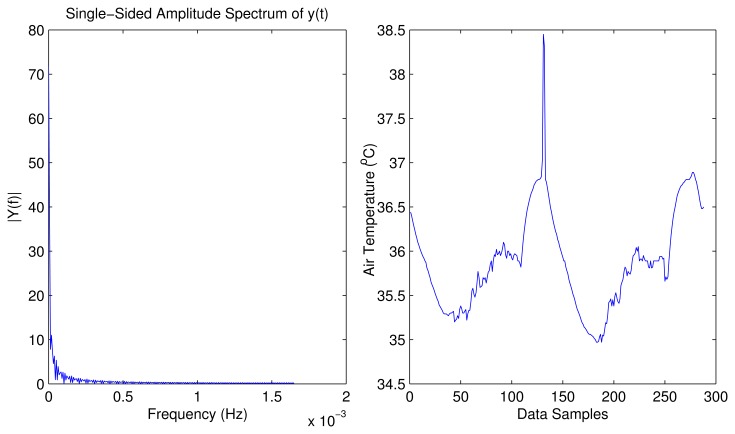
Compressibility of wildfire data-trace.

To verify the suitability of SWIFTNET to wildfire data-trace, we examine the compressibility of the signal shown is Figure 5. We perform a numerical experiment on the time-domain of the data trace. Suppose we represent as a vector *f* ∈ ℝ*^n^*, assume x ∈ (image)*^n^* is the Fourier transform of *f*. We choose the *k* largest elements of *x* and zero all other *n* − *k* elements, and put them into vector *y*. We then perform an inverse FFT on the vector *y* to get the resulting vector *v*, which is a recovered version of the original signal. We can see that from Figure 5 almost all the energy of the time-domain signal is concentrated on a few largest Fourier coefficients of its interval-domain transform. From this figure, it is seen that the signals from the wildfire environment are compressible or sufficiently sparse. Based on this experiment, we can safely apply SWIFTNET to the wildfire application scenario, without losing much accuracy on the reconstructed signal.

## 5. Evaluation and Results

### 5.1. Wireless Monitoring Platform

**Figure 6:**
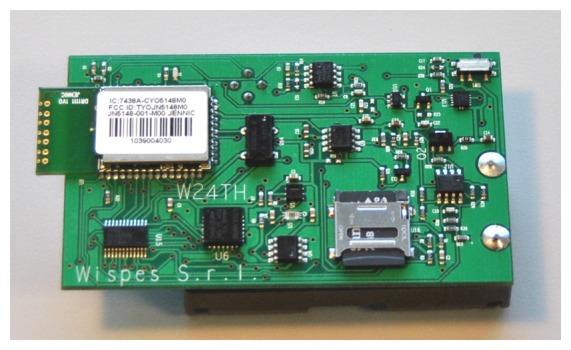
A front view of a WISPES W24TH node prototype.

The SWIFTNET algorithm has been implemented on WISPES W24TH nodes, shown in Figure 6. It is based on the Jennic microprocessor [60], one of the few 32-bit architectures available in the market with a power consumption comparable to a TelosB [61]. It is equipped with 128 KB RAM, 512 KB FLASH with 2.4 GHz radio transceiver (IEEE802.15.4 compliant) and Zigbee Pro compliant [62]. It utilizes an aggressive power management method using 8 μA in sleep mode, 17 mA in transmit mode, and 23 mA in receive mode; this guarantees a longer battery lifetime. The details of the hardware design and the stack are presented in [63]. A dual client control platform is used that consists of MATLAB for the high-end reconstruction algorithm and the CACTI [64] a powerful network graphic tool for visualization, online data management, and storage.

### 5.2. Real Test-Bed Implementation of SWIFTNET

**Figure 7:**
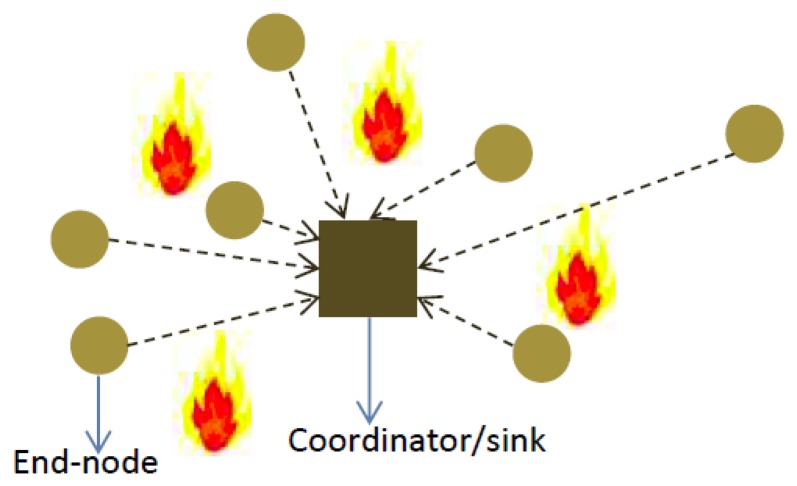
Experimental testbed topology.

The efficacy of SWIFTNET is tested with air temperature data, using our in-house deployment testbed of 15 sensor nodes powered by batteries, shown in Figure 7 for our application scenario. The nodes are organized in a star topology, each node acquire its data and runs the SWIFTNET algorithm. For every sample interval, a node decides to transmit or compress the data depending on the threshold value that is set. Although, nodes are multi-modal, meaning they could sense other parameters such as humidity, wind speed, *etc.*, we prefer to concentrate on the air temperature, as it offers easy testing on the reactiveness of our testbed. We implemented SWIFTNET algorithm on the WISPES W24TH nodes, and the pseudo-code is given in Algorithm 2. Each leaf node runs this algorithm. Upon startup, a node initializes its parameters on Lines 2 and 3, and it starts sensing the phenomenon. The sensed data is acquired at every data collection round in Line 4. Then, the algorithm intelligently switches between CS and adaptive prediction at every data collection round, and the alarm condition is checked for violation. If the sensed data is up to a dangerous level predefined by the user on Line 5, it calls the prediction algorithm on Line 6, which is built on an adaptive sampling strategy in Equation (15), and it triggers an immediate reporting of the phenomenon depending on the rate of change of the air temperature, in this case, a wildfire application. From our experiments, on average the reporting fluctuates between 12 to 30 s. When the air temperature drops below the threat level, the node calls the CS algorithm shown in Lines 9 to 24, and thus proceeds with data compression depending on the size of the history table. When the history table is full, the data is transmitted in one or a few packets. This way the radio usage is significantly minimized, since it only turns “ON” when transmitting. For example the *comp*_(_*_vector_*_)_ is transmitted to the sink node, which uses Algorithm 1 for the decompression and online distributed inference.



**Algorithm 2** SWIFTNET algorithm.
1:Call Task START2:Initialize parameters (LFSR seed)3:Parameters: *Count, comp*_(_*_vector_*_)_, *CS*_(_*_vector_*_)_, *SizeofHistorytable*4:Call Task *sense*_(_*_data_*_)_5:**if** (*sense*_(_*_data_*_)_ ≥ *ρ*) **then**6: Call adaptive prediction algorithm7:**else**8:re-Initialize sample interval9: *CS*_(_*_vector_*_)_ ← sense_(_*_data_*_)_10: Call Task Sleep11: **if** Count ≥ SizeofHistorytable **then**12:  **for** (*i in* 1 : *Sizeofcomp*_(_*_vector_*_)_) **do**13:   buffer ← 014:   **for** (*j in* 1 : *SizeofHistorytable*) **do**15:     buffer ← rand() × *CS*_(_*_vector_*_)_ [j]16:   **end for**17:   *comp*_(_*_vector_*_)_[i] ← buffer;18:  **end for**19:  Call Task Transmit *comp*_(_*_vector_*_)_20:  Call Task Sleep21:  re-initialize LFSR with seed22:  Count ← 023: **end if**24: Count++25:**end if**


### 5.3. Experimental Setup and Evaluation

To realize the experimental testbed, we set up 15 WISPES nodes to communicate within a cluster to the sink node. Note that, it is possible to extend the cluster network into a hierarchical topology as investigated in [53], in which, every cluster-head performs aggregation by summing up the compressed vectors. In our study, we assume the sink is rich in energy-resource due to unlimited power supply, and it is capable of processing the data received from the network (see [63], for detailed discussion on the hardware unit and the software protocol stack).

Considering that we are dealing with a fire scenario, we are constrained to mimic a real wildfire by generating an artificial fire prescribed as test-burn with the use of a heat gun. The heat gun is capable of generating a regulated hot air up to 200 ^°^C in a short burst. This enables testing of SWIFTNET across different temperature ranges and on several nodes. Particularly, we conducted two separate scenarios; an equivalent of a 24 h and a 48 h test-burns. For both cases, the air temperature is sampled at 5 min intervals, which amounts to 288 samples in 24 h and 576 samples in 48 h, the sparsity K is set to 25% of the total samples collected in each case.

**Figure 8:**
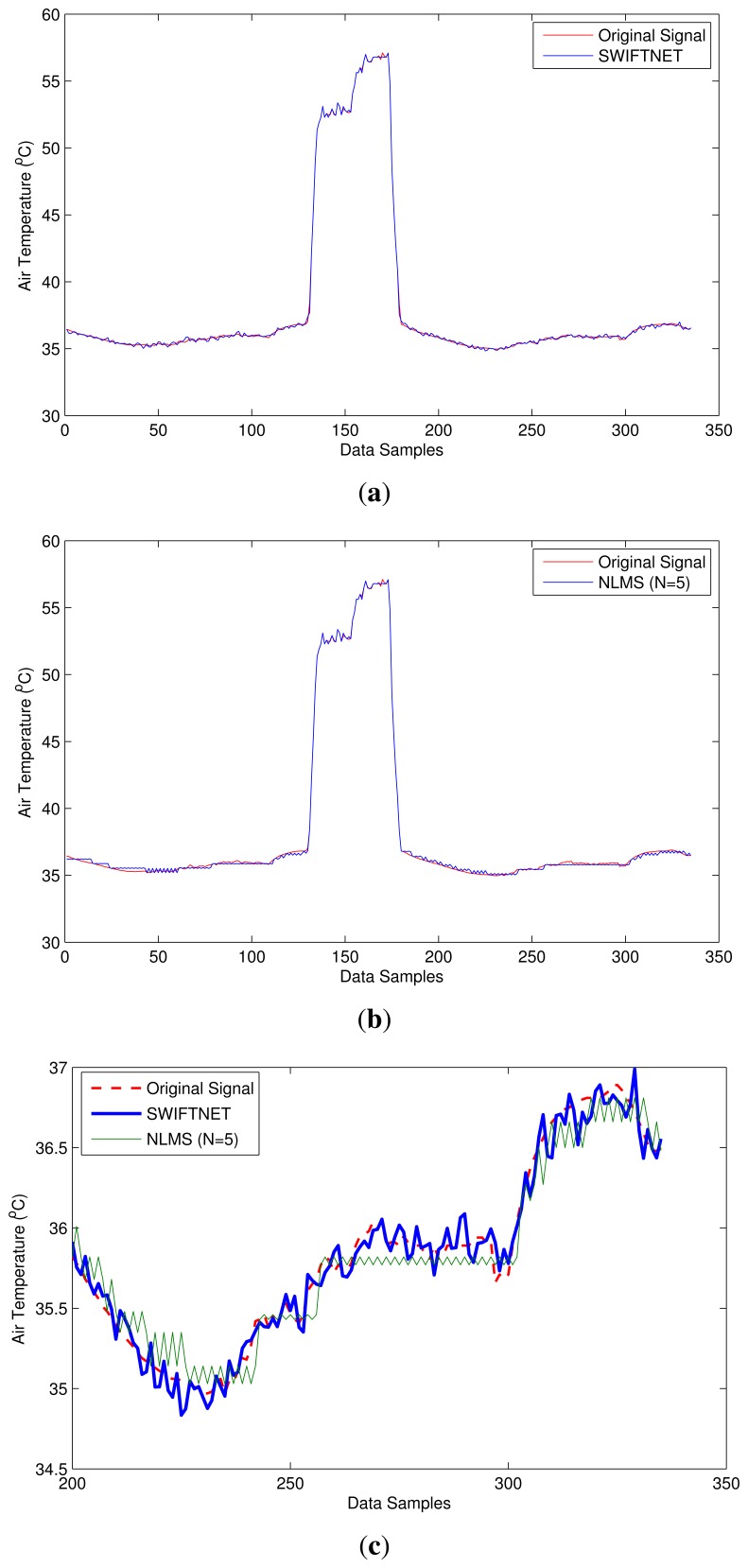
Signal reconstruction from Node 1, for a 1-day wildfire monitoring test-burn using a 40 ^°^C *threshold* cut-off point. The sparsity rate K is set to 25% at 5 min sampling interval: (**a**) Example SWIFTNET data trace; (**b**) Example data trace from default adaptive prediction method; (**c**) A snapshot of real and predicted sensor readings.

The first test-burn offers a glimpse on the behavior of SWIFTNET with respect to acquiring data aggressively when there are meaningful events and otherwise performs data compression. Figure 8a depicts how SWIFTNET begins operation in the normal mode roughly between 0 and 142 data points when aggressive data gathering is not necessary, and then it adaptively switches to fire zone (recall from Figure 4) at about 143 to 185 data points, when the user-defined *threshold* is consistently violated over the *n*-window period. We observed the operation worked seamlessly in the network and all the nodes that captured the changes in air temperature reported their data accurately. SWIFTNET in Figure 8a transmitted less data to the sink compared with the adaptive prediction (NLMS) in Figure 8b, which transmitted more data to the sink. However, not much accuracy is lost by SWIFTNET, the details are presented under the discussion of result section.

**Figure 9:**
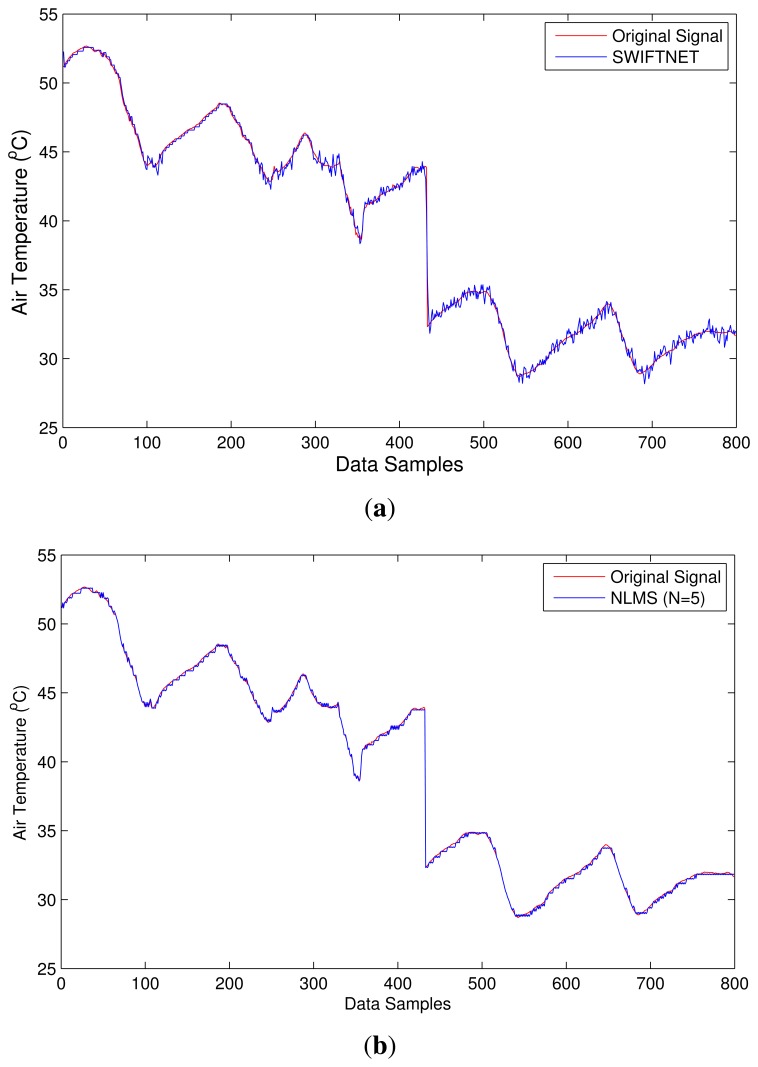
Signal reconstruction from Node 2, for a 2-day wildfire monitoring test-burn using a 45 ^°^C *threshold* cut-off point. The sparsity rate K is set to 25% at 5 min sampling interval: (**a**) Example SWIFTNET data trace. Fire is generated at different time intervals to test the efficacy of SWIFTNET to different operational mode; (**b**) Example data trace from default adaptive prediction method.

The second test-burn, that is the 2-day period offers a more interesting scenario, as we are able to generate fire scenarios at different time intervals of the days using a threshold cut-off air temperature of 45 ^°^C (this is shown in Figure 9a). This figure reveals different fire intensities between 0 and 300 data points during the test-burn and then the evolution of the air temperature as the fire cools off. It can be observed from this figure that our algorithmic solution switches back and forth between data compression and adaptive prediction seamlessly without disrupting the operation of the nodes in the network. For example, roughly between data points 80 and 120, the air temperature drops below the 45 ^°^C threshold and the node automatically switches to CS. Beyond 120 data point we increase the burst of the hot air temperature, and the node switches back to adaptive prediction. This trend can be observed on other data points on the series. Comparing this behavior between SWIFTNET in Figure 9a and adaptive prediction algorithm (NLMS) in Figure 9b, it can be observed that the adaptive prediction algorithm achieves smoother trend following the original data trace. However, this is at the expense of a higher transmission cost. Instead, SWIFTNET is able to transmit less data with a comparable level of accuracy. More details of this result is discussed in the next sections. An important characteristic is that the nodes are able to work autonomously, while sending their individual data packets to the sink node for the signal reconstruction.

Overall, both Figures 8 and 9, we can observe the differences between the curves of SWIFTNET and NLMS. In particular, the NLMS curves are much smoother than the SWIFTNET curves due to the reason that, in SWIFTNET, only *K* elements are randomly selected during the data acquisition process. This signal is further compressed using the online generated LFSR matrix. According to the CS theory, the larger the value of *K*, the smoother would be the reconstructed signal, *i.e.*, the curve. The choice of *K* in this instance could depend on how often the radio of the nodes would be kept in “ON” mode to allow data acquisition. Keeping the radio in this state for a longer period during data acquisition could mean more energy would be consumed on each node, hence a tradeoff between the accuracy of the signal reconstruction and the energy consumption in the deployed system.

Next, we briefly examine the choice of the prediction models used for the wildfire application.

#### 5.3.1. Prediction Model Consideration

Here we examine what model is suitable for a wildfire monitoring environment, considering that there are several candidate prediction models as examined in literature [4] (pp. 163–170), namely: (1) Replicated models such as Kalman filters, normalized least-mean square, autoregressive models, *etc.*; (2) Aggregative models such as, average model, distributed regression, *etc*. Recently, one of the main discussions in literature [65] on prediction models has been centered around its implementation on the node platforms. A common drawback of prediction models is that they require prehistorical data for the model build-up process, which makes it computationally expensive for a node to implement, especially due to memory limits, and hence they are not often feasible to implement. The mode of operation requires the sink node to build a model and to broadcast the model parameters to the entire network. We can easily see that if the model buildup process fails, either due to the constraint of a noisy wireless channel, the entire network will fail. Even if a model is successfully transmitted in the network, when the model ceases to properly fit the data, re-learning a new model would be time consuming for an application that requires high responsiveness, such as in wildfire disaster. The delays associated with gathering the training dataset could result in loss of lives and property.

To better understand the environment for a wildfire scenario, when there is a fire disaster, there is a high probability that the wildfire characteristics, such as the intensity of the fire, location, sensing needs, duration of the burn, *etc.*, will be different from the previous fire disasters. Hence, it is insufficient to use existing datasets to build *a priori* an efficient prediction model for the future fire disasters. Also, since fast responsiveness is highly desirable in such an application, preliminary gathering of datasets to build a complex model would be undesirable. Based on these considerations, and due to the nature of the wildfire events, models such as multi-variate Gaussian [32], Dual Kalman Filter (DKF) [33], Autoregressive Integrated Moving Average (ARIMA) [34] and the likes are therefore not effective choices. Similarly, other studies such as [66] proposed to use neural network models, however, such models are complex to implement on a resource-limited sensor node. Hence, they are not suitable choice on our platform. The candidate prediction model should be computationally inexpensive, and should report readings that are “outliers” with high responsiveness. Models such as Normalized Least Mean Squares (NLMS) [49] and Fixed-Weighted Moving Averages (WMA) [39] (p. 8) are good candidates for a wildfire monitoring application. Due to the results of the comparative study conducted in [65] on data prediction models, we chose NLMS as the prediction model, together with our proposed adaptive sampling strategy; note that the default adaptive prediction method is used interchangeably as NLMS. Furthermore, the choice of NLMS is motivated by its simplicity and ease of implementation on our platform. The NLMS model, while simple, still offers excellent accuracy and high data reduction in WSNs for some event-based monitoring applications.

#### 5.3.2. Performance Metric

The performance metric used to assess our method is defined below:

- **Reactivity**: This is the measure of how much the protocol is able to react to changes in the monitored phenomenon. This implies the adaptability of the sample interval to the changes in the signal.
- **Relative Update Rate** (*R_update_*): This is the ratio of the number of transmissions using SWIFTNET to the number of transmitted packets, when using a default adaptive prediction algorithm for the entire monitoring period. The relative update rate for the default adaptive prediction model is 1, since all updates will be sent when required. It is expected that the relative update rate, *R_update_*, is bounded as 0 ≤ *R_update_* ≤ 1; the lower this value is, the better the gain in terms of energy savings.
- **Accuracy**: The accuracies of the methods are measured using both the Relative Error (RE) and the Mean Absolute Error (MAE) metrics. This reflects the accuracy of the reconstructed samples with respect to the corresponding observation.

#### 5.3.3. Reactivity of SWIFTNET

**Figure 10:**
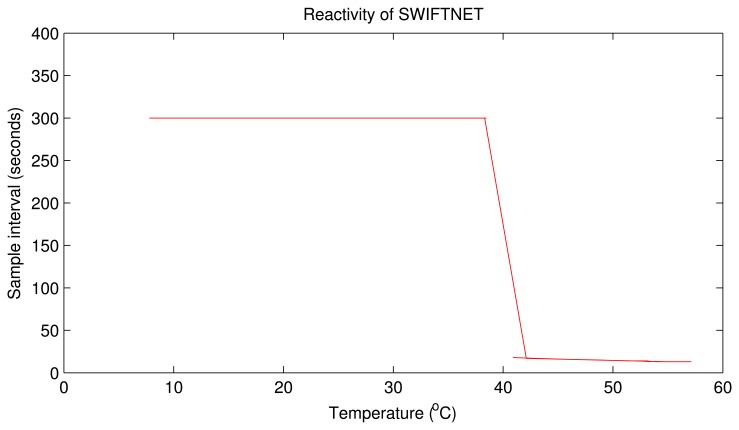
Dynamic sampling interval of SWIFTNET.

Figure 10 depicts the reactivity of SWIFTNET using a real-world data trace from one of our experiments. It can be seen that the sampling interval remains fixed at 300 s during the CS data acquisition phase, and changes immediately there is a fire event signal, sensed at 40 ^°^C and above. The sampling interval automatically adapts to the signal; in this case, the sampling interval increases and fluctuates between 12 and 30 s. Similar performance on the reactivity of SWIFTNET is observed from the other nodes. We expect that this behavior should be valid for a larger network, since our algorithm design is distributive. Consequently, all nodes can adaptively adjust their sampling interval, according to the respective phenomenon that they monitor. It is worth mentioning here that, other adaptive sampling approaches such as [67] can also be used on top of the prediction algorithm. However, we believe that our approach is suitable for the wildfire application use-case, and on our stack architecture framework. An open research direction in this area could be to conduct a comparative study on the different adaptive sampling strategies on top of the prediction method for different application setups. The objective could be to find the optimal performance that can be achieved with respect to the reactiveness and fault-tolerance. This could serve as a guide to WSN developers and researchers in the field.

### 5.4. Discussion of Results

The aim of this article is to design an energy-efficient light-weight protocol that can significantly limit the unnecessary transmission often associated with WSN deployments, with the main goal of minimizing the overall energy consumption in the network. From a previous work in [63], which examined the implication of packet transmission in a WSN that uses the W24TH node prototype. The result is that one packet transmission suppressed is equivalent to approximately 13 min of added life-time for each node. Clearly, any data acquisition protocol that can limit packet transmission without missing useful events would be preferable. We analyzed SWIFTNET and the default adaptive prediction algorithms in this regard.

From Figure 8a, Node 1 reveals the signal trace captured by SWIFTNET; when the fire event is generated and throughout the operation, 47 samples are collected in addition to the 288 samples that are acquired and compressed over an equivalent period of 24-hours at 5 min sampling interval. In total, 335 samples are logged on our CACTI database. Similarly, in Figure 9, Node 2 collected 223 samples during the fire event in addition to the 576 samples over a 48-hour period; this gives a total of 799 samples logged. From these figures, using SWIFTNET, Node 1 transmitted 33 packets out of the 335 packets and Node 2 transmitted 85 packets out of the 799 packets to the sink node for data gathering and processing.

The same phenomenon using the default adaptive prediction algorithm as shown in Figure 8b and Figure 9b. From these figures, Node 1 transmitted 68 packets out of 335 packets and Node 2 transmitted 271 packets out of 799 packets respectively.

In Figure 8c we show overlapping plots for both SWIFTNET and NLMS (default adaptive prediction) over a range of data points extrapolated from Figure 8a,b. The graphs reveal how the two methods are able to follow the evolution of the signal trace. Although, the signal trace looks less chaotic, SWIFTNET is still able to follow the evolution with the exhibition of few noises in the signal reconstruction. If the goal is to further improve the accuracy beyond 1% error bound, this generated signal trace from SWIFTNET could be smoothed out using extrapolation method. Overall, the prediction error for NLMS is within the user defined error bound *ϵ_max_*. Similarly, the accuracy of SWIFTNET is not significantly different from the performance of the NLMS.

By comparing the performance of SWIFTNET and the default adaptive prediction from the two Figures, from Node 1 SWIFTNET achieved (a) an *R_update_* ≈ 0.48, which fractionally represents an improvement of more than half, with RE of 0.0071 and (b) MAE of 0.2723 compared with the default adaptive prediction having RE of 0.0059 and MAE of 0.231. Although, the accuracy of the default adaptive prediction is slightly better than SWIFTNET, the default adaptive prediction transmitted more data to achieve that level of accuracy. We believe that the difference in accuracy is not significant compared to the amount of energy saved by using SWIFTNET.

**Figure 11:**
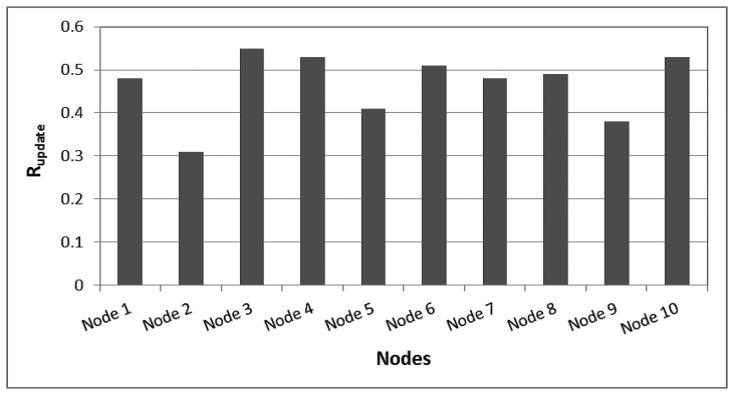
Relative update rate (*R_update_*) of SWIFTNET taken from 10 nodes.

Similarly, from Node 2, SWIFTNET shown in Figure 9 achieved an *R_update_* ≈ 0.31, RE of 0.0071 and MAE of 0.271 compared with the default adaptive prediction having *R_update_* of 1, RE of 0.0069 and MAE of 0.2717. Overall, both the energy saving and accuracy are in favor of SWIFTNET and comparatively, the difference in accuracy is not significant. Figure 11 and Table 1 show an average summary result comparing SWIFTNET with the default adaptive prediction method. The results are taken from 10 nodes chosen from our testbed. On average, SWIFTNET achieved more than 50% improvement over the default adaptive prediction method used without loss in accuracy. Both Δ*RE* and Δ*MAE* reflect accuracy loss or gain of SWIFTNET relative to the default adaptive prediction. A minus, *i.e.*, “−” value, indicates SWIFTNET's loss in accuracy relative to the default adaptive prediction.

### 5.5. Limitations of the Approach

One of the few limitations of our experimental set-up is that, to test the efficacy of SWIFTNET, the fire sources are generated using the heat gun. The reservation here would be that the heat gun fire source does not exhibit exactly the same intensity as in a real wildfire scenario, in which case the rate of change of the signal amplitude may be high within a short period of time. In addition, in the event of intense wildfire disasters, the air temperature might be very high (over 100 ^°^*C*); as a consequence, a few of the nodes could be physically damaged due to the very high temperature and could result to packet loss. To avoid this, it is envisaged that a very good thermal protective cover could be used in a dense deployment, such that packet loss from a few nodes would not greatly impact the information gathering process. Moreover, we acknowledge that the experiments have been conducted on a small testbed of a few nodes. In the future, it is our intention to extend this work to cover several nodes, while using a different application setup, such as micro-changes in climate condition in a conservatory or museum.

## 6. Conclusions

This article is anchored on the improvements offered by a combination of data compression, prediction and adaptive sampling strategies, to achieve a robust intelligent data acquisition process in WSNs. We have proposed and implemented a light-weight data acquisition algorithm, termed SWIFTNET, for an event-detection monitoring system. One of the main contributions of this work is that we have successfully employed a mix of two major fields of data-driven methods, (1) a data reduction method, and (2) an energy-efficient data compression method. Both categories have been widely used separately in various domains, as surveyed in [15]. However, we have provided a holistic approach that combines a mix of efficient algorithms in these categories, to further optimize the energy consumption in a real-world wireless sensor network deployment. This is achieved by building an adaptive prediction method as a layer on top of a compressed sensing algorithm.

Unfortunately, attempts to optimize energy efficiency are often in conflict with the goals of network reactiveness for alarms and urgent messages. Thus, we presented SWIFTNET, a data acquisition protocol for a reactive network application. A wildfire monitoring application has been presented as a use-case for our design framework, to test the performance of SWIFTNET in a real-world deployment testbed.

The design method and implementation are presented, followed by the performance analysis. In summary, the performance of SWIFTNET proved to significantly improve the network lifetime of a WSN deployment compared with a default adaptive prediction approach. On average, over 50% improvement is achieved in terms of data transmission, which translates to energy savings, with no loss in accuracy over the default method widely referenced in the literature. We note that, although, the signal sources used are nearly sparse or exhibit an average sparsity rate, it is possible to reconstruct the original signal using SWIFTNET at a desirable level of accuracy without significant loss.

In conclusion, this study reveals that the network lifetime of a WSN deployment can be significantly prolonged by employing an algorithmic approach such as SWIFTNET. This algorithm design incurs minimal cost, and requires no prior existing knowledge of the monitoring phenomenon. Hence, it is easily scalable for very large deployments that are envisaged to span into decades, requiring little or no human intervention. An interesting future direction for this study would be to test the capability of SWIFTNET on a larger network comprising hundreds of nodes, and with respect to different application scenarios, such as monitoring drastic changes in micro-climate conditions indicating occupancy in a building. Similarly, a performance test of SWIFTNET on different network topologies could be an interesting direction. However, we leave these topics as open research directions for the future.

## Figures and Tables

**Table 1:** Performance summary of SWIFTNET: Result from 10 nodes, Δ*MAE* and Δ*RE* represent the relative accuracy differences for SWIFTNET compared with the default adaptive prediction method, where “−” signifies that SWIFTNET has a lower accuracy.

**Node**	**Δ*MAE***	**Δ*RE***
1	−0.0413	−0.0012
2	0	−0.0002
3	0.082	0.0021
4	−0.0312	−0.0047
5	0.075	0.0018
6	−0.011	−0.0017
7	−0.0659	−0.0062
8	0.019	0.0013
9	0.070	0.0016
10	−0.015	−0.0023
Average	0.00816	−0.00095
